# "The Lancet" and Hospital Nurses

**Published:** 1890-08-09

**Authors:** 


### Passing Topics.

### "THE LANCET" AND HOSPITAL NURSES.

The preliminary report of the *Lancet* Commissioner is chiefly noticeable for good intentions and somewhat meagre results. We scarcely needed the labours of a special Commissioner continued over a year to demonstrate that many nurses are overworked, and many more complain of the shortness of the yearly holiday, bad cooking, and a want of variety in the diets. These things sound familiar enough, and not less so because they have all come up quite recently during the proceedings "in another place." Whether the method adopted by the Commissioner has helped to bring about this paucity of information is a matter for individual opinion. We incline to the belief that the very deferential letter addressed to "as many nurses of the London hospitals as I have been able to reach by means of private introductions," was scarcely calculated to ensure the fullest result, while the limitation of the inquiry to those nurses whom the Commissioner could approach by means of private introductions appears unwise, partly because by this self imposed restriction the Commissioner handicapped his own powers, and partly, because the notion likely to be engendered by the letter that the nurse was asked to confer a personal favour by answering the questions addressed to her, appears to receive additional encouragement and sanction.

The representative of the *Lancet* when engaged upon a public and wholly commendable task, might well have spared himself an apologetic attitude towards those in whose interests the labour was undertaken. It could not fail to increase the reticence, which might have been entirely overcome under more bracing treatment. Our nurses are not deficient in courage, but, like other people who have embarked upon a venture, they are not wholly insensible to atmospheric conditions, and when they find the pilot so conscious of a threatening storm that he speaks with an ominous hush upon his lips, lest a loud word should cause the clouds to burst, they naturally take to whispering too, or else are silent.

But here criticism ends, and we congratulate the *Lancet* upon its action, forestalled though it may seem to be by the Lords' Committee. It is well it should be known that there are hospitals where the nurses are expected to scrub the floors, to clean lavatories, and perform other wholly menial offices, and we quite agree with the protest of the Commissioner against such practices, both as wrong in themselves and as "very bad economy."

The question of the food is as important as any raised. There is abundant concurrent testimony, besides that accumulated by the gentleman whose report we are considering, to show but too much room for improvement. It is obvious that a nurse, more perhaps than anybody else, scarcely excepting the sick ones she tends, needs to find her appetite stimulated. A laborious occupation usually induces desire for food, but the nurse sees much and does much calculated to take away rather than strengthen appetite, and it is easy to suspect that the anæmia in nurses, of which we hear so much, is due to semi-starvation. Their appetites revolt at the food placed before them, not perhaps because of its quality, but merely because it may not be served in a tempting way and is too often the same one day as another.

It is unfortunate that everything is commonly left to routine and the cook. What is wanted, and we write after many years of experience, is intelligent catering by an officer set free from the miserable thraldom of the diet sheets, possessed of a healthy appetite, and who, having no real or pretended contempt for feeding, does not consider it derogatory or tiresome to give thought and attention day by day to every detail, and whose native sense is keen enough to grasp the elementary fact that a home—nay a community—may be ruined by a too perfunctory performance of duty by a housekeeper or her equivalent. Agricola.

